# The risk of developing diabetes during antipsychotic drug treatment: A nationwide study among 31,856 patients with schizophrenia

**DOI:** 10.1192/j.eurpsy.2023.339

**Published:** 2023-07-19

**Authors:** N. Marker Madsen, O. Köhler-Forsberg, C. Garne Rohde, A. Aalkjær Danielsen, O. Mors

**Affiliations:** 1 Aarhus University Hospital Psychiatry, Aarhus, Denmark; 2 Aarhus University Hospital Psychiatry, Aarhus

## Abstract

**Introduction:**

Antipsychotics (AP) are the primary pharmacological treatment for schizophrenia but increase the risk for diabetes, with recent meta-analyses indicating important differences between specific APs. However, these findings are based on randomized clinical trials, which only include 20% of patients seen in everyday clinical settings, and are hence prone to selection bias.

**Objectives:**

We aim to investigate 1) the actual risk of developing diabetes in patients treated with APs using real-world data and 2) whether there are risk differences between specific APs.

**Methods:**

We conducted a retrospective cohort study using Danish nationwide healthcare registers. We identified all individuals receiving a schizophrenia diagnosis from January 1, 1999, to January 1, 2019 and an age- and sex-matched reference population from the general population. The primary outcome was diabetes, identified via hospital discharge diagnoses and redeemed prescriptions for glucose-lowering drugs. First, we compared the risk of developing diabetes between patients with schizophrenia and the age- and sex-matched reference population. Second, among the patients with schizophrenia, the association between AP drug treatment and the risk of diabetes were analyzed. Third, risk differences for developing diabetes between specific AP drugs were investigated. We used cox regression for all analyses, the latter adjusted for age, sex, year of schizophrenia diagnosis, Charlson Comorbidity Index (CCI), occupational status, marital status, education and schizophrenia severity (use of antipsychotics, antidepressants, anxiolytics/hypnotics/sedatives, mood stabilizers and number of psychiatric admissions in the year preceding the diagnosis of schizophrenia).

**Results:**

We identified 31,856 patients with schizophrenia and 159,280 reference individuals. Patients with schizophrenia had an increased risk of developing diabetes compared with the reference individuals (unadjusted HRR: 3.12, 95%CI: 2.98-3.28). Treatment with AP in patients with schizophrenia, compared to periods with no AP use, was associated with an increased risk of developing diabetes (adjusted HRR: 2.04, 95%CI: 1.75-2.38). This risk was particularly increased among individuals treated with lurasidone (HRR: 2.66, 95%CI: 1.10-6.42), sertindole (HRR: 2.10, 95%CI 1.51-2.93), paliperidone (HRR: 1.84, 95%CI 1.47-2.31), clozapine (HRR: 1.74, 95%CI 1.49-2.03) and aripiprazole (HRR: 1.54, 95%CI 1.35-1.75), whereas treatment with zuclopenthixol (HRR: 1.00, 95%CI 0.82-1.23), flupentixol (HRR: 0.71, 95%CI 0.40-1.25) and pimozide (HRR: 0.74, 95%CI 0.31-1.78) were not associated with an increased risk of diabetes.

**Conclusions:**

This real-world study indicates differences in the risk of developing diabetes between specific AP compounds. Further analyses will be presented at the conference.

**Disclosure of Interest:**

None Declared

